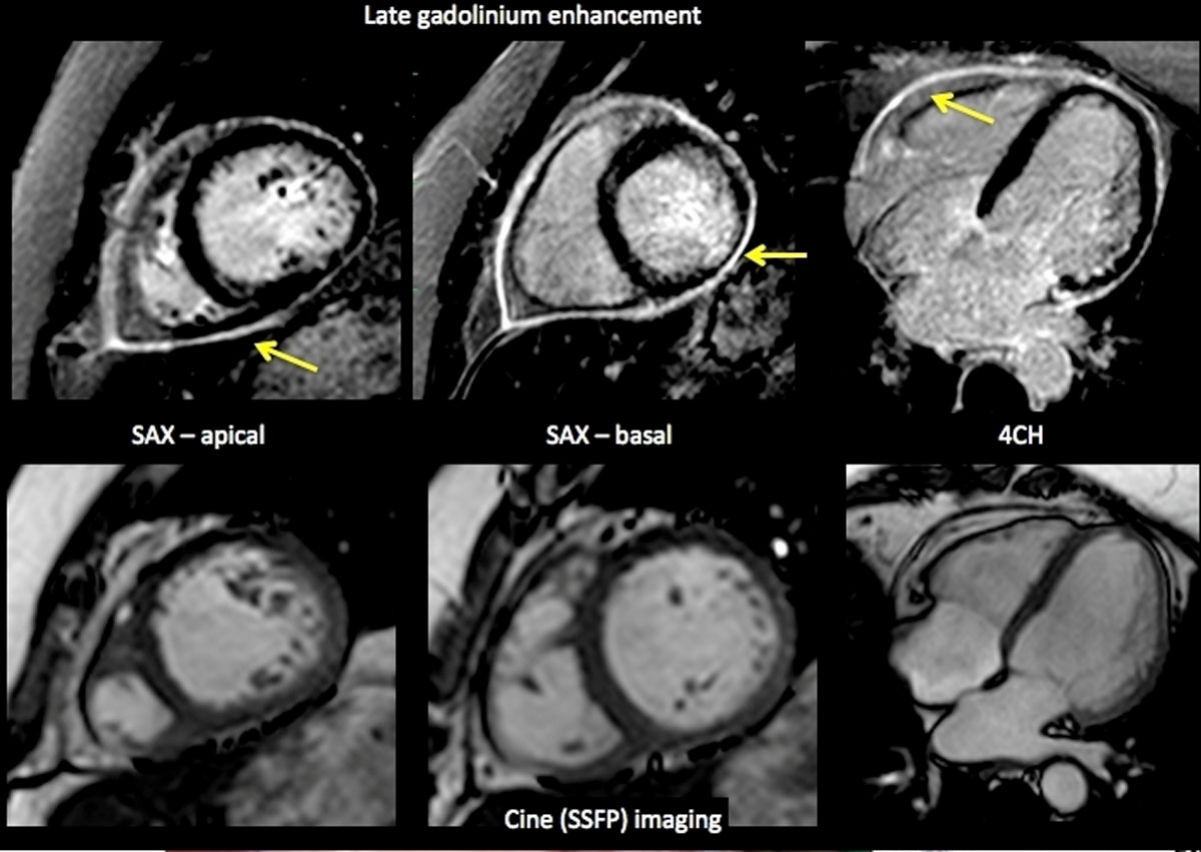# Native T1 mapping by cardiovascular resonance imaging detects subclinical cardiomyopathy in patients with systemic lupus erythematosus

**DOI:** 10.1186/1532-429X-15-S1-O22

**Published:** 2013-01-30

**Authors:** Valentina O Puntmann, David D'Cruz, Zachary Smith, Ana Pastor, Peng Choong, Gerald Carr-White, Tobias Voigt, Shirish R Sangle, Tobias Schaeffter, Eike Nagel

**Affiliations:** 1 Cardiovascular Imaging, King's College London, London, UK; 2 The Lupus Unit, King's College London, London, UK; 3 Philips Innovative Technologies, Clinical Research, London, UK; 4 Department of Medical Physics and Bioengineering, King's College London, London, UK

## Background

Increased inflammation has been linked to myocardial dysfunction and heart failure. We investigated whether T1 mapping by cardiovascular magnetic resonance (CMR) in patients with systemic lupus erythematosus (SLE) can detect subclinical cardiac involvement.

## Methods

A total of 37 SLE female patients subjects (mean age 41±11years) underwent CMR for routine assessment of cardiac perfusion, function and scar. T1 mapping was performed in single short axis slice before and 15 minutes after gadolinium administration.

## Results

Sixteen age-matched subjects from the clinical pool with a low pre-test probability and normal CMR acted as a control group. Both groups had normal volumes and global systolic function (ejection fraction (%): control vs. SLE group: 60±13 vs. 56±12, p=0.57) and similar LV mass index (LVmass index (g/ m2): 43±12 vs. 51±12 g/ m2, p=0.18). SLE patients had significantly reduced longitudinal strain (%, -19±4 vs. -16±3, p=0.05) and showed intramyocardial (70%) or pericardial (63%) late gadolinium enhancement. SLE patients had significantly increased preconstrast myocardial T1 (T1native ) values and lambda (p<0.01). In comparison of significant measures, we identified T1native as the strongest discriminator between the patients and controls.

## Conclusions

We demonstrate that in SLE patients without significant coronary artery disease there is evidence of subclinical perimyocardial involvement. Among variables to discern the subclinical cardiomyopathic process, T1 mapping performs best to discriminate between health and disease.

## Funding

National Institute for Health Research (NIHR) comprehensive Biomedical Research Centre award.

**Figure 1:**
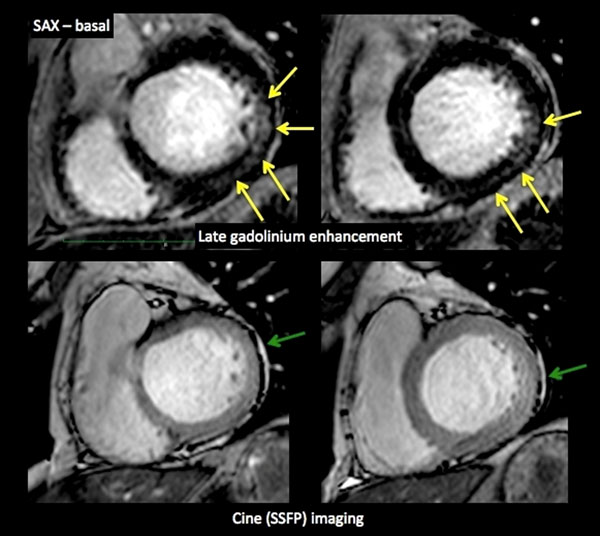


**Figure 2:**